# Stem-Loop I of the Tembusu Virus 3′-Untranslated Region Is Responsible for Viral Host-Specific Adaptation and the Pathogenicity of the Virus in Mice

**DOI:** 10.1128/spectrum.02449-22

**Published:** 2022-10-10

**Authors:** Li Mao, Yu He, Zhen Wu, Xiaoli Wang, Jiaqi Guo, Senzhao Zhang, Mingshu Wang, Renyong Jia, Dekang Zhu, Mafeng Liu, Xinxin Zhao, Qiao Yang, Sai Mao, Ying Wu, Shaqiu Zhang, Juan Huang, Xumin Ou, Qun Gao, Di Sun, Anchun Cheng, Shun Chen

**Affiliations:** a Institute of Preventive Veterinary Medicine, Sichuan Agricultural University, Chengdu, Sichuan, China; b Research Center of Avian Disease, College of Veterinary Medicine, Sichuan Agricultural University, Chengdu, Sichuan, China; c Key Laboratory of Animal Disease and Human Health of Sichuan Province, Sichuan Agricultural University, Chengdu, Sichuan, China; Wuhan Institute of Virology

**Keywords:** TMUV, 3'UTR, SLI structure, host-specific adaptation, *in vitro* proliferation, viral pathogenicity in mice

## Abstract

Tembusu virus (TMUV), an avian mosquito-borne flavivirus, was first identified from Culex tritaeniorhynchus in 1955. To validate the effects of the 3′-untranslated region (3′UTR) in viral host-specific adaptation, we generated a set of chimeric viruses using CQW1 (duck strain) and MM 1775 (mosquito strain) as backbones with heterogeneous 3′UTRs. Compared with rMM 1775, rMM-CQ3′UTR (recombinant MM 1775 virus carrying the 3′UTR of CQW1) exhibited enhanced proliferation *in vitro*, with peak titers increasing by 5-fold in duck embryonic fibroblast (DEF) cells or 12-fold in baby hamster kidney (BHK-21) cells; however, the neurovirulence of rMM-CQ3′UTR was attenuated in 14-day-old Kunming mice via intracranial injection, with slower weight loss, lower mortality, and reduced viral loads. In contrast, rCQ-MM3′UTR showed similar growth kinetics *in vitro* and neurovirulence in mice compared with those of rCQW1. Then, the Stem-loop I (SLI) structure, which showed the highest variation within the 3′UTR between CQW1 and MM 1775, was further chosen for making chimeric viruses. The peak titers of rMM-CQ3′UTRSLI displayed a 15- or 4-fold increase *in vitro*, and the neurovirulence in mice was attenuated, compared with that of rMM 1775; rCQ-MM3′UTRSLI displayed comparable multiplication ability *in vitro* but was significantly attenuated in mice, in contrast with rCQW1. In conclusion, we demonstrated that the TMUV SLI structure of the 3′UTR was responsible for viral host-specific adaptation of the mosquito-derived strain in DEF and BHK-21 cells and regulated viral pathogenicity in 14-day-old mice, providing a new understanding of the functions of TMUV 3′UTR in viral host switching and the pathogenicity changes in mice.

**IMPORTANCE** Mosquito-borne flaviviruses (MBFVs) constitute a large number of mosquito-transmitted viruses. The 3′-untranslated region (3′UTR) of MBFV has been suggested to be relevant to viral host-specific adaptation. However, the evolutionary strategies for host-specific fitness among MBFV are different, and the virulence-related structures within the 3′UTR are largely unknown. Here, using Tembusu virus (TMUV), an avian MBFV as models, we observed that the duck-derived SLI of the 3′UTR significantly enhanced the proliferation ability of mosquito-derived TMUV in baby hamster kidney (BHK-21) and duck embryonic fibroblast (DEF) cells, suggesting that the SLI structure was crucial for viral host-specific adaptation of mosquito-derived TMUVs in mammalian and avian cells. In addition, all SLI mutant viruses exhibited reduced viral pathogenicity in mice, indicating that SLI structure was a key factor for the pathogenicity in mice. This study provides a new insight into the functions of the MBFV 3′UTR in viral host switching and pathogenicity changes in mice.

## INTRODUCTION

In nature, interspecies transmission is a common phenomenon among many different types of viruses, including influenza virus and coronavirus (1–3). Flaviviruses are a large group of arborviruses within the *Flaviviridae* family that are vectored to birds or mammals principally by arthropods and are divided into four ecological groups: mosquito-borne flaviviruses (MBFVs), tick-borne flaviviruses (TBFVs), insect-specific flaviviruses (ISFVs), and no known vector flaviviruses (NKFVs) (4–6). Tembusu virus (TMUV) is a newly emerging mosquito-borne epornitic flavivirus that can cycle between mosquito and avian hosts. In 1955, the first mosquito-derived TMUV strain, MM 1775, was isolated in Malaysia from Culex tritaeniorhynchus (7). There were no reports on TMUV infecting avians until 2000, when Sitiawan virus was the first TMUV strain found to cause encephalitis and retard growth in broiler chickens (8). Later, in 2010, TMUV broke out in southeast China, and since then it has caused severe economic losses in the duck industry and aroused public concern (9, 10). However, the molecular mechanisms of the cross-species transmission of TMUV from mosquito to avian remain unclear.

Naturally, TMUV infection occurs with a large host spectrum but is limited in a closed cycle involving adult mosquitoes as vectors and avians (e.g., ducks, chickens, and geese) as reservoirs. However, experimental evidence indicated that Kunming and BALB/c mice can be infected with TMUV by intracranial inoculation, causing severe neurological symptoms or even death (11, 12). Besides, TMUV replicates well in many kinds of mammalian cell lines (e.g., baby hamster kidney [BHK-21] cells, Vero cells, HeLa cells, and 293T cells) (13). In addition, an investigation of duck industry workers indicated its potential transmission from ducks to humans (14). Therefore, the latent emergence of strains virulent in humans should be carefully considered.

**FIG 1:**
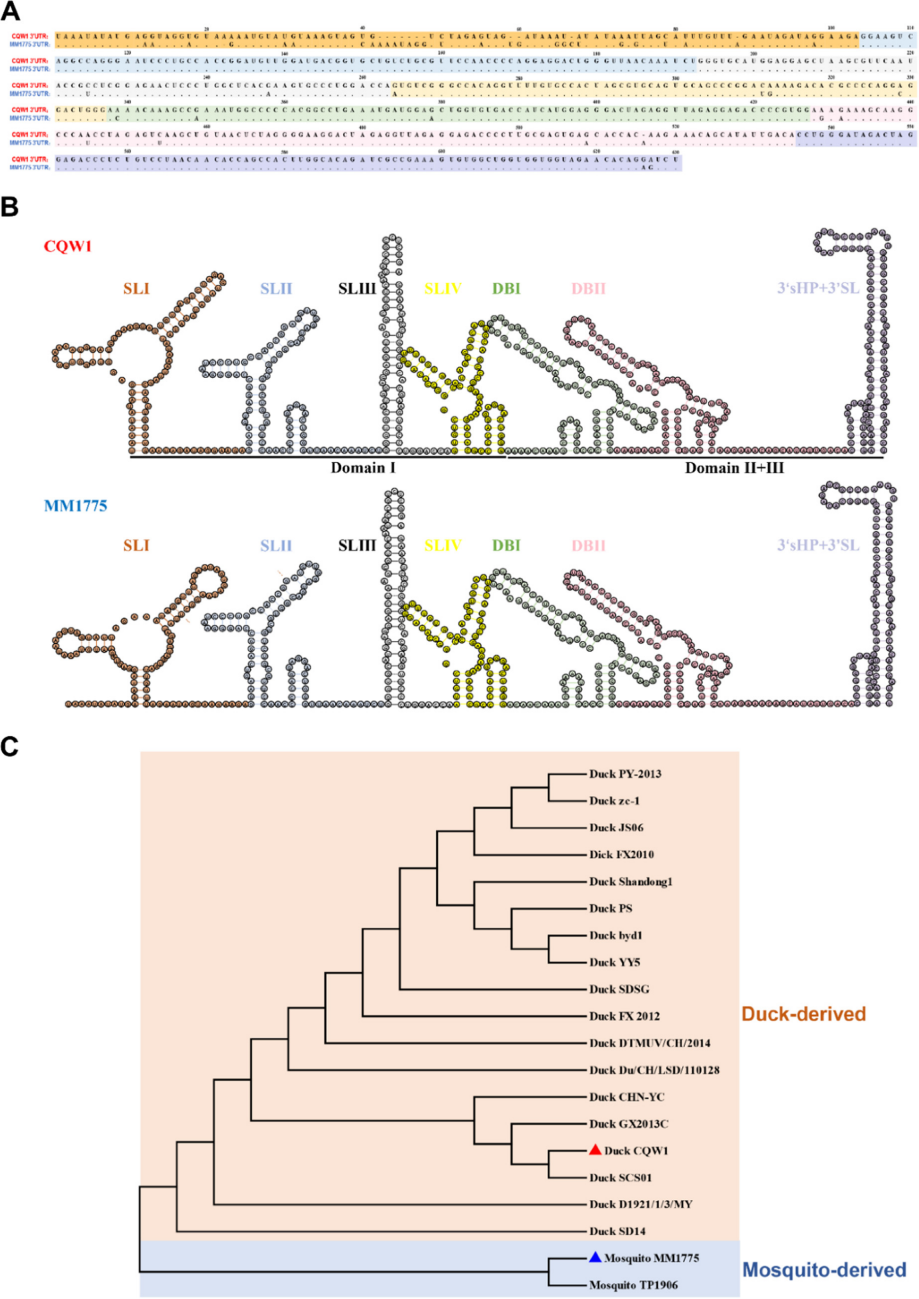
Characteristics of the TMUV 3′-untranslated region (3′UTR). (A) Structure-based sequence alignment between CQW1 and MM 1775 3′UTR performed with ClustalW algorithms; nucleotides within different RNA secondary structures are sequentially highlighted with different background colors, a hyphen (-) indicates a lack of base, and a dot (.) indicates a conservation of base. (B) RNA secondary structure of the CQW1 (top) and MM 1775 (bottom) 3′UTRs predicted using the RNA fold web server or mFold web server and modified artificially. The 3′UTR is divided into three domains, including four stem-loop structures (SLI to SLIV) in domain I, two dumbbell structures (DBI and DBII) in domain II, and a 3′ small hairpin (3′sHP) + 3′SL in domain III. (C) Phylogenetic analysis of the TMUV 3′UTR. The phylogenetic tree was constructed based on the TMUV 3′UTR sequences using MEGA7 software with the neighbor-joining (NJ) method. The reference sequences of the viruses were obtained from the GenBank database.

The RNA genome of TMUV is single-stranded and approximately 11 kb in length, with a unique open reading frame (ORF) that is flanked by a 5′-untranslated region (5′UTR) and a 3′UTR. The ORF encodes a polyprotein that is processed into three structural proteins (C, prM, and E) and seven nonstructural proteins (NS1, NS2A, NS2B, NS3, NS4A, NS4B, and NS5) by viral and host proteases (15). An intriguing characteristic of the 3′UTR in the flavivirus genome is the presence of highly structured and duplicated RNA secondary structures (16). In the case of TMUV, the 3′UTR includes four nearly analogous stem-loop structures (SLI to SLIV) in the variable region domain I, two identical dumbbell elements (DBI and DBII) in the moderately conserved region domain II, a small hairpin (sHP), and the 3′ terminal stem-loop (3′SL) in the most conserved region domain III (Fig. 1B). Many studies have revealed that flavivirus 3′UTRs possess a number of essential functions in viral replication, host-specific adaptation, and pathogenicity. Cyclization of the flavivirus genome represents the initiation of viral replication and is mediated by means of long-range RNA-RNA interactions between elements, including the upstream AUG region, the downstream AUG region, and the cyclization sequence at the 5′ and 3′UTRs (17). Duplicated RNA secondary structures mapped in the flavivirus 3′UTRs play opposite roles in viral replication in different host cells; while deleting the SLII in Dengue virus (DENV) or SLI in Zika virus (ZIKV) provides a great replication advantage in mosquito cells, the two SLs appear to play redundant functions in human cells (18, 19). The nucleotide mutations in the Japanese encephalitis virus (JEV) SLIV and DBI structures contribute to pathogenicity differences in mice (20). In addition, the flavivirus 3′UTR is also related to subgenomic RNA generation and viral transmission (21, 22).

In previous studies, we observed that the mosquito-origin TMUV rMM 1775 and the duck-origin TMUV rCQW1 exhibited significant differences in multiplication *in vitro* (23, 24). To further identify the role that the TMUV 3′UTR played in proliferation *in vitro* and the pathogenicity of the virus for mice, a series of chimeric viruses with heterogeneous 3′UTRs were generated based on the reverse genetic method using CQW1 or MM 1775 as the backbone. The *in vitro* growth characteristics of these chimeric viruses were determined in BHK-21, duck embryonic fibroblast (DEF), and C6/36 cells, and the virulence was evaluated in 14-day-old Kunming mice by intracranial infection. Our data revealed that the TMUV SLI structure within the 3′UTR was responsible for viral host-specific adaptation of the mosquito-derived strain in avian and mammalian cells and for regulating viral pathogenicity in 14-day-old mice, which provided a new molecular mechanism for viral host switching and changes in viral pathogenicity in mice.

## RESULTS

### TMUV rCQW1 and rMM 1775 exhibited significant proliferation differences *in vitro*.

**FIG 2:**
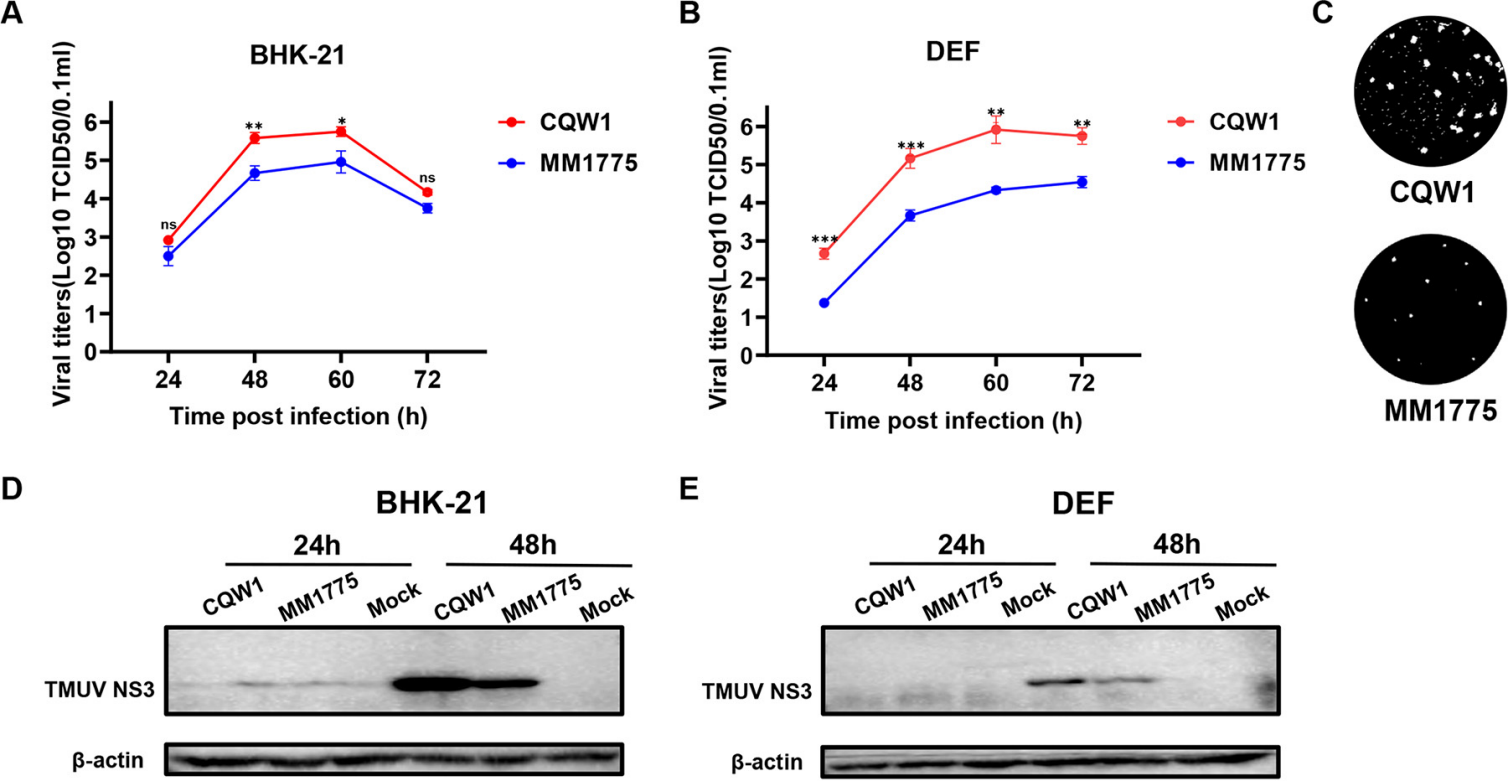
*In vitro* characteristics of rCQW1 and rMM 1775. Comparison of the growth kinetics of rCQW1 and rMM 1775. (A, B) Baby hamster kidney (BHK-21) cells (A) and duck embryonic fibroblast (DEF) cells (B) were infected with rCQW1 or rMM 1775 at 250 median tissue culture infectious dose (TCID_50_) in 24-well plates, and viral titers were measured at the indicated time points using the TCID_50_ method in BHK21 cells. The data represent the means and standard deviations (SDs) of three independent cell cultures and were tested for statistical significance using Student’s *t* test. *, *P* < 0.05; **, *P* < 0.01; ***, *P* < 0.001; ns, no significance. (C) Plaque phenotypes of rCQW1 and rMM 1775. The virus-infected BHK-21 monolayers were incubated under an overlay medium containing 1% methyl cellulose and then stained with crystal violet at 6 days postinfection (dpi). (D, E) BHK-21 cells (D) and DEF cells (E) were infected with rCQW1 or rMM 1775 at 300 TCID_50_ and harvested at the indicated time points, and the synthesis of viral NS3 proteins was detected using a mouse anti-DTMUV-NS3 polyclonal antibody. TMUV, Tembusu virus.

In our previous study, we successfully rescued a duck-derived TMUV rCQW1 and a mosquito-derived TMUV rMM 1775 by reverse genetics technology (23, 24). First, we compared the viral proliferation characteristics of these two strains *in vitro*. In BHK-21 cells, rMM 1775 and rCQW1 shared comparable replication trends, and at 60 hpi, both reached peak titers, but at 48 and 60 h, the titers of rMM 1775 were 8- and 7-fold lower than those of rCQW1 (Fig. 2A). This result suggested that rMM 1775 showed a weaker replication ability than rCQW1 in BHK-21 cells. This finding was more obvious in DEF cells; at each time point, the titers of rMM 1775 were significantly lower than those of rCQW1, and the peak titer was 38-fold lower at 60 hpi (Fig. 2B). Then, we measured plaque morphology, and the result was as expected: the plaque size of rMM 1775 was smaller than rCQW1 in the BHK-21 cell monolayer (Fig. 2C), suggesting lower infectivity of rMM 1775 compared with rCQW1. Correspondingly, we also assessed the expression level of viral nonstructural protein 3 (NS3). The results showed that in both BHK-21 and DEF cells, the accumulation of viral NS3 protein was lower for rMM 1775 than for rCQW1 at 48 hpi (Fig. 2D and E). Moreover, our previous study indicated that rMM 1775 showed stronger replication ability than rCQW1 in C6/36 cells (24). Altogether, these data suggested that TMUV rCQW1 and rMM 1775 exhibited significant proliferation differences *in vitro*; rCQW1 exhibited higher viral titers and NS3 expression levels in both BHK-21 and DEF cells and stronger infectivity in BHK-21 cells but weaker proliferation in C6/36 cells.

### rMM 1775 showed stronger virulence in mice than rCQW1.

**FIG 3:**
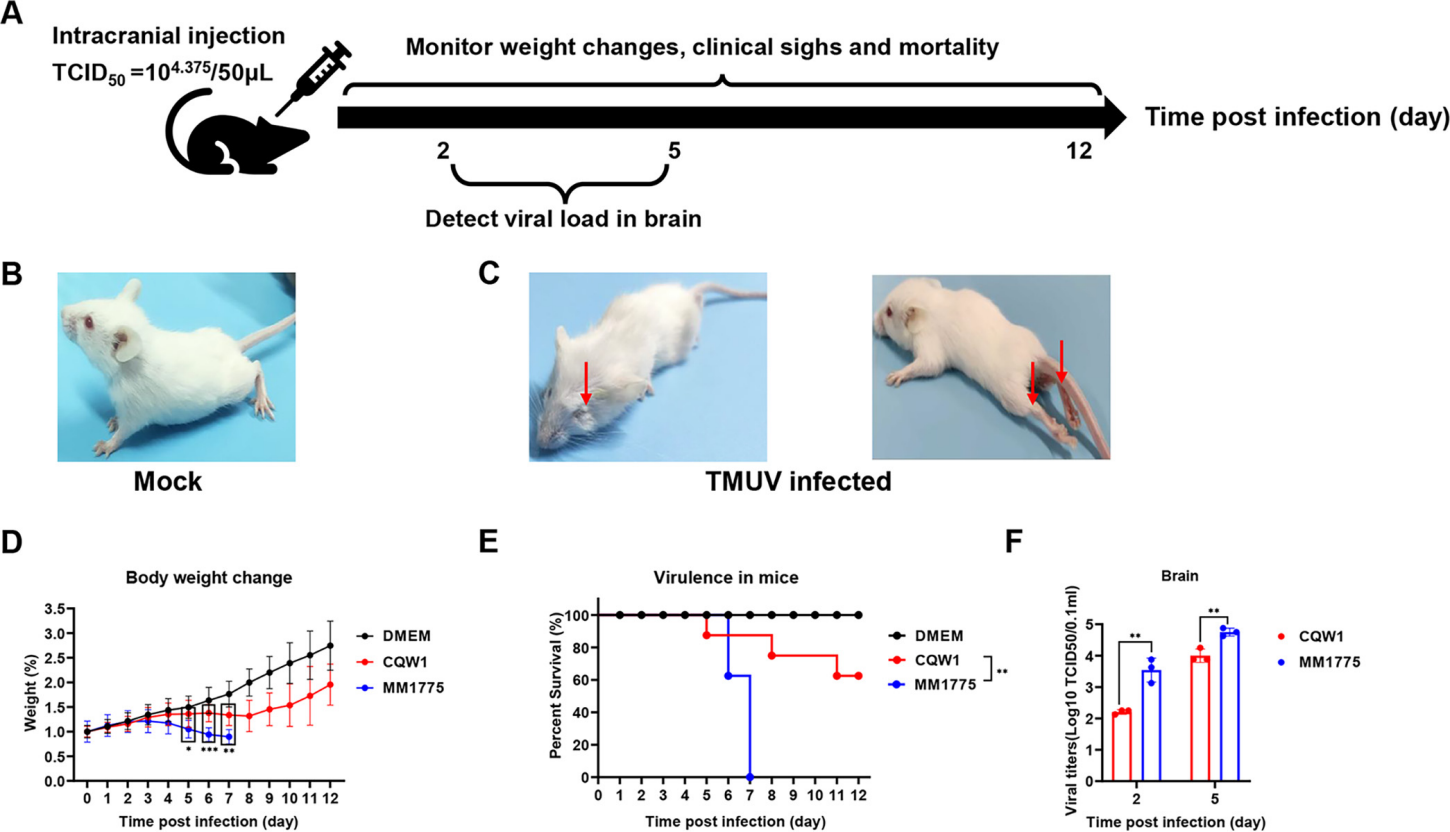
Pathogenicity of rCQW1 and rMM 1775 in mice. (A) Mouse experimental design. Fourteen-day-old Kunming mice were intracranially injected with rCQW1 or rMM 1775 at 10^4.375^ TCID_50_/50 μL, respectively, and weight changes, clinical signs, and mortality were monitored daily. The viral load was detected at 2 and 5 dpi. (B, C) While mice in the mock group remained healthy (B), mice infected with rCQW1 or rMM 1775 became blind, paralyzed (C), and even died. (D) Weight changes postinfection (days 0 to 12). Statistical significance at each time point was analyzed using multiple *t* tests with the Holm-Sidak method, and the data with no significance are not shown as “ns.” (E) Survival curves of mice in the rCQW1- and rMM 1775-infected groups. Statistically significant differences in survival were analyzed using the log-rank (Mantel-Cox) test. (F) Viral titers in the brain at 2 and 5 dpi. The data represent the means and SDs of three independent individuals and were tested for statistical significance using Student’s *t* test. Statistical significance: *, *P* < 0.05; **, *P* < 0.01. DMEM, Dulbecco’s modified Eagle’s medium.

Previous studies have shown that Kunming mice can be infected with TMUV by intracranial inoculation (12). To measure the difference in the pathogenicity of the virus in mice between rMM 1775 and rCQW1, 14-day-old Kunming mice were infected with 50 μL (median tissue culture infectious dose [TCID_50_] = 10^4.375^/50 μL) of rMM 1775 or rCQW1 by intracranial injection (Fig. 3A). While mice in the mock group remained healthy (Fig. 3B), typical clinical symptoms were produced such as depression, feather messiness, blindness, paralysis (Fig. 3C), and some death in both rMM 1775- and rCQW1-infected mice. However, the mice in the rMM 1775-infected group exhibited a more obvious weight decrease and significantly higher mortality than rCQW1-infected mice (Fig. 3D and E). In this case, mice inoculated with rCQW1 began to lose weight at 5 days postinfection (dpi) and recovered from 9 dpi, and the mortality reached 40%. In contrast, mice inoculated with rMM 1775 began to lose weight earlier at 3 dpi and never recovered, and all mice died within 7 days. Moreover, we also detected the viral load in the mouse brain, and the results showed that the titers of mice in the rMM 1775-infected group were significantly higher than those in the rCQW1-infected group at 2 and 5 dpi, showing 21- and 6-fold increases, respectively (Fig. 3F). All of these data indicated that compared with rCQW1, rMM 1775 showed stronger virulence in mice by intracranial infection, exhibiting more severe weight loss and significantly higher mortality and viral load in the brain.

### Sequence alignment and RNA secondary structure analysis of the TMUV 3'UTR.

Many studies have shown that the specialization of flavivirus 3′UTR sequences or duplicated RNA secondary structures is associated with host adaptation (16, 18, 25). Due to the large proliferation differences *in vitro* or virulence in mice between duck-derived TMUV rCQW1 and mosquito-derived TMUV rMM 1775, we analyzed the nucleotide and RNA secondary structure differences in the viral 3′UTR. Sequence alignment showed that the nucleotides within most RNA secondary structures of the 3′UTR were highly conserved, showing more than 90% similarity, except for the SLI structure, which presented only 66.98% similarity (Fig. 1A). Meanwhile, RNA secondary structure predictions indicated that while the SLI element was highly variable, other secondary structures were highly conserved (Fig. 1B). These results suggested that the SLI structure might be important for viral host switching. Furthermore, we also measured the evolutionary relationship of different TMUV strains. A phylogenetic tree was constructed based on the complete 3′UTR sequences of different TMUVs. The results suggested that the genetic relationship of TMUV was related to the initial isolated hosts (Fig. 1C). According to these analyses, we speculated that the TMUV 3′UTR might be a crucial factor that promotes transmission of the virus from mosquitoes to ducks.

### rMM 1775 was more sensitive to the 3′UTR substitution in terms of viral proliferation *in vitro*.

**FIG 4:**
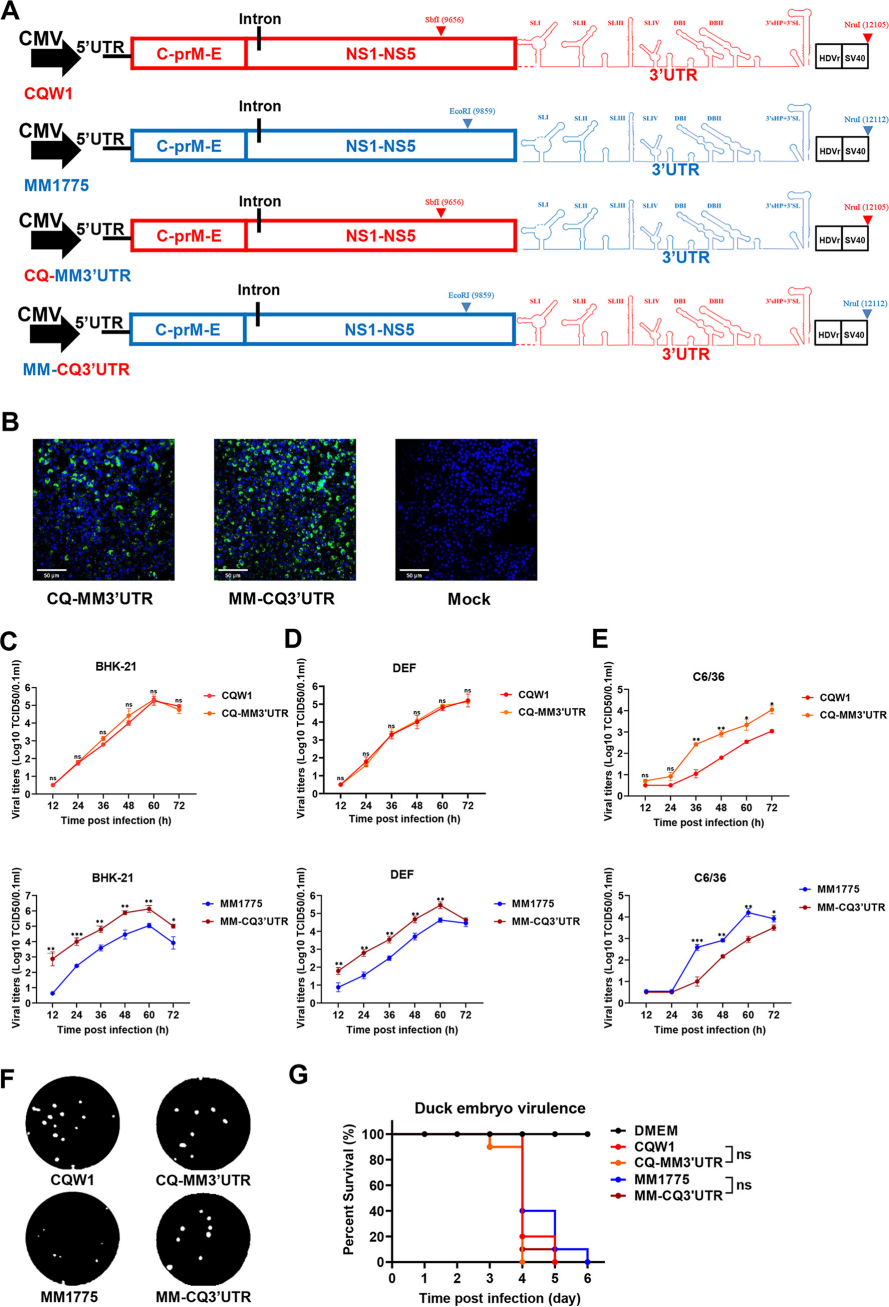
*In vitro* characteristics of 3′UTR chimeric TMUV viruses. (A) Schematics of the construction of 3′UTR chimeric viruses based on the infectious clones pACNR-CQW1-Intron and pACNR-MM 1775-Intron. The corresponding mutational fragments were engineered by a series of overlapping PCRs, and rational enzyme sites were chosen at the indicated positions. (B) Recovery of 3′UTR chimeric viruses confirmed by indirect immunofluorescence assay (IFA) in BHK-21 cells. (C to E) Growth kinetics of parent viruses and 3′UTR chimeric viruses were determined in BHK-21 cells (C), DEF cells (D), and C6/36 cells (E) at 250 TCID_50_. The data represent the means and SDs of three independent cell cultures and were tested for statistical significance using Student’s *t* test. (F) Plaque phenotypes of parent viruses and 3′UTR chimeric viruses. The virus-infected BHK-21 monolayers were incubated under an overlay medium containing 1% methyl cellulose and then stained with crystal violet at 6 dpi. (G) Virulence of the parent viruses and 3′UTR chimeric viruses in duck embryos (*n* = 10/group) at 1,000 TCID_50_. The statistical significance of survival was analyzed using a survival curve and the log-rank (Mantel-Cox) test. Statistical significance: *, *P* < 0.05; **, *P* < 0.01; ***, *P* < 0.001; ns, no significance. CMV, cytomegalovirus.

Large variations were observed between the 3′UTR of CQW1 and MM 1775. Thus, we hypothesized that the TMUV 3′UTR might be pivotal for viral multiplication in different hosts. To dissect this possibility, two chimeric recombinant viruses (rCQ-MM3′UTR and rMM-CQ3′UTR) were generated by exchanging the viral 3′UTR using CQW1 or MM 1775 as the backbone (Fig. 4A) and were rescued successfully, as confirmed by IFA (Fig. 4B) and sequencing. Next, we measured the growth kinetics in different host cells. The results showed that in both BHK-21 and DEF cells, rCQ-MM3′UTR (recombinant CQW1 carrying the 3′UTR from MM 1775) displayed similar proliferation ability compared with rCQW1, whereas rMM-CQ3′UTR (recombinant MM 1775 carrying the 3′UTR from CQW1) exhibited enhanced growth ability compared with rMM 1775. In this regard, the peak titers increased 5- fold in BHK-21 cells (Fig. 4C) and 12-fold in DEF cells (Fig. 4D). Interestingly, in C6/36 cells, the result was quite the opposite: rCQ-MM3′UTR grew faster than rCQW1, while rMM-CQ3′UTR grew slower than rMM 1775 (Fig. 4E). Concurrently, we also compared their plaque morphology, and the results showed that while rCQW1 and rCQ-MM3′UTR exhibited comparable plaque sizes, rMM-CQ3′UTR produced larger plaques than rMM 1775 (Fig. 4F). The results indicated that rCQW1 and rCQ-MM3′UTR might possess similar infectivity, and rMM-CQ3′UTR might be more infectious than rMM 1775. These results suggested that MM 1775 was more sensitive to changes in the viral 3′UTR. In addition, we also evaluated viral virulence in 9-day-old duck embryos via allantoic cavity inoculation (Fig. 4G). After incubation with 1,000 TCID_50_ of the chimeric viruses or parent viruses, all of the duck embryos in each group died within 4 to 6 dpi, and no significant difference was observed.

Altogether, these data suggested that the TMUV 3′UTR had a great influence on viral proliferation in different host cells. The MM 1775 3′UTR substitution hampered rCQW1 proliferation in mosquito cells (C6/36) but did not change proliferation in other cells. Moreover, CQW1 3′UTR substitution significantly improved rMM 1775 growth ability in both BHK-21 and DEF cells but was shown to be decreased in mosquito cells.

### Substitution of the 3′UTR significantly attenuated the pathogenicity of rMM 1775 in mice.

**FIG 5:**
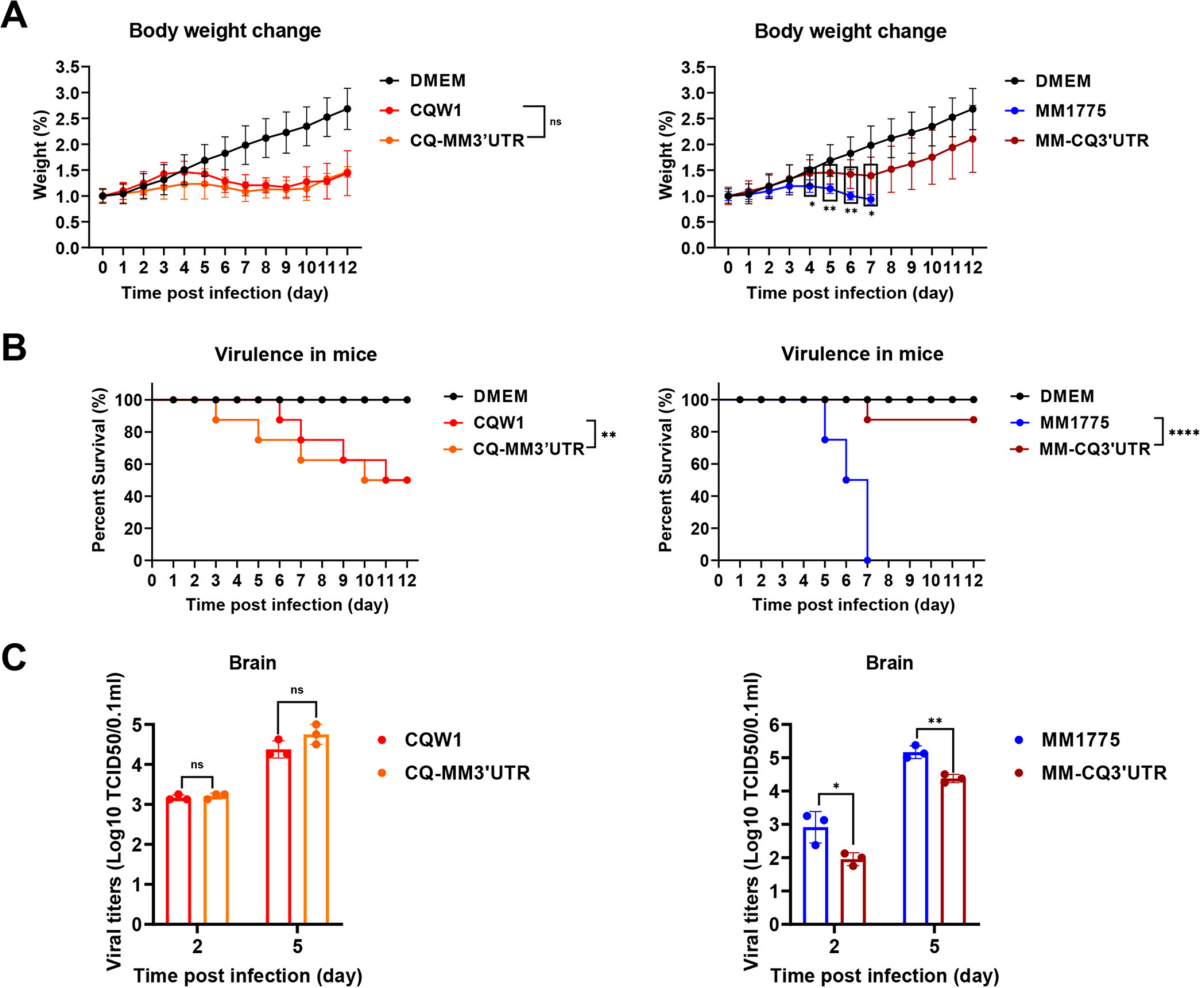
Pathogenicity of 3′UTR chimeric viruses in mice. Fourteen-day-old Kunming mice were intracranially injected with parent viruses or 3′UTR chimeric viruses at 10^4.375^ TCID_50_/50 μL, respectively. (A) Weight change postinfection (days 0 to 12). Statistically significant differences in weight change at each time point were analyzed using multiple *t* tests with the Holm-Sidak method. (B) Survival curves of mice. Statistically significant differences in survival were analyzed using the log-rank (Mantel-Cox) test. (C) Viral titers in mouse brains at 2 and 5 dpi. The data represent the means and SDs of three independent individuals and were tested for statistical significance using Student’s *t* test. Statistical significance: *, *P* < 0.05; **, *P* < 0.01; ****, *P* < 0.0001; ns, no significance.

To investigate the effect of viral 3′UTR substitution on viral pathogenicity in mice, 14-day-old Kunming mice were infected with parent viruses or 3′UTR chimeric recombinant viruses at 10^4.375^TCID_50_/50 μL by intracranial inoculation, and weight changes, clinical signs and mortality were recorded daily. While the weight changes of mice in the rCQW1- and rCQ-MM3′UTR-infected groups showed the same trend, mice in the rMM 1775- and rMM-CQ3′UTR-infected groups exhibited visible differences, especially at 5 to 6 dpi, in this regard (Fig. 5A). Correspondingly, the mouse mortality presented a similar conclusion: mice in the rCQW1- or rCQ-MM3′UTR-infected groups had 50% mortality (4 of 8); in contrast, mice in the rMM 1775-infected group reached 100% mortality (8 of 8), and only 12.5% mortality (1 of 8) was observed in the rMM-CQ3′UTR-infected group (Fig. 5B). In addition, the viral loads in the mouse brain were also measured by the TCID_50_ method. As expected, the titers of rCQW1 or rCQ-MM3′UTR in mouse brains reached the same level at 2 and 5 dpi. However, the titers of rMM 1775 were 9- and 6-fold higher than those of rMM-CQ3′UTR at 2 or 5 dpi, respectively (Fig. 5C), which was significantly different. These results indicated that MM 1775 bearing the CQW1 3′UTR substitution significantly attenuated its virulence in 14-day-old mice in terms of weight changes, mortality, and viral loads in brain but not vice versa.

### SLI was a crucial structure for the proliferation improvement of rMM 1775 *in vitro*.

**FIG 6:**
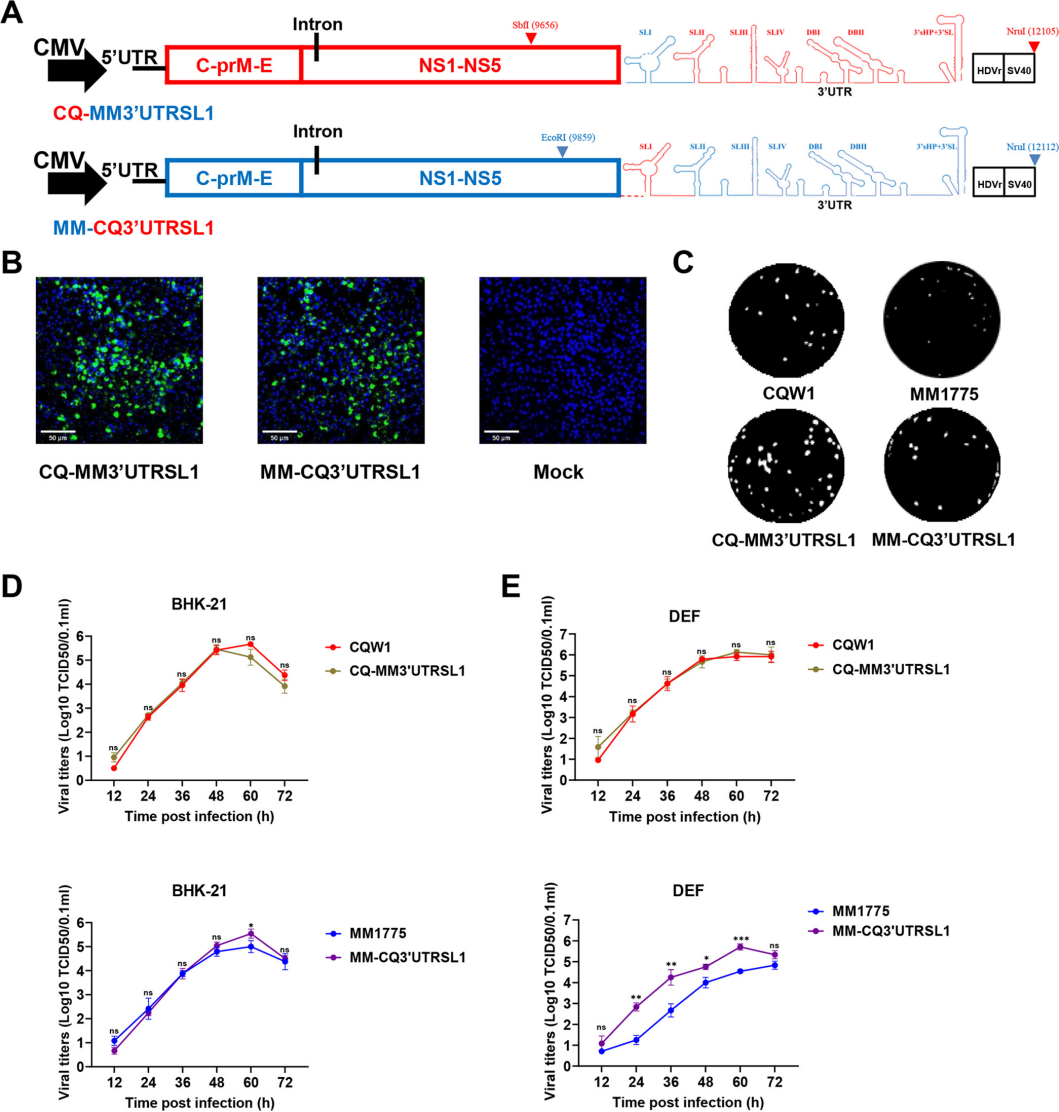
*In vitro* characteristics of SLI chimeric viruses. (A) Schematics of the construction of SLI chimeric viruses. (B) Recovery of SLI chimeric viruses confirmed by IFA in BHK-21 cells. (C) Plaque phenotypes of parent and SLI chimeric viruses. The virus-infected BHK-21 monolayers were incubated under an overlay medium containing 1% methyl cellulose and then stained with crystal violet at 6 dpi. (D, E) Growth kinetics of parent and SLI chimeric viruses were determined in BHK-21 cells (D) and DEF cells (E) at 250 TCID_50_. All data represent the means and SDs of three independent cell cultures and were tested for statistical significance using Student’s *t* test. *, *P* < 0.05; **, *P* < 0.01; ***, *P* < 0.001; ns, no significance.

As mentioned above, the TMUV 3′UTR contains three domains with four stem-loop structures, two dumbbell structures, and a unique 3′SL structure. Among these, SLI was the most variable in both nucleotides and RNA secondary structures. Thus, we constructed SLI chimeric recombinant viruses (rCQ-MM3′UTRSLI and rMM-CQ3′UTRSLI) (Fig. 6A) to study the function of the SLI element in viral host switching and pathogenicity in mice. The viruses were successfully rescued based on transfection into BHK-21 cells and confirmed by IFA (Fig. 6B) and sequencing. Plaque morphological examination showed that rCQW1 and rCQ-MM3′UTRSLI produced comparable plaques. Moreover, rMM-CQ3′UTRSLI produced larger plaques than rMM 1775 (Fig. 6C). We further examined the viral growth kinetics, and the results showed that rCQW1 and rCQ-MM3UTR SLI displayed similar growth adaptation in both BHK-21 cells and DEF cells (Fig. 6D and E), whereas at 60 hpi, rMM-CQ3′UTRSLI exhibited enhanced growth adaptation in BHK-21 cells, showing a 4-fold higher titer (Fig. 6D). This proliferation increase was more obvious in DEF cells. From 24 to 60 h, the titers of rMM-CQ3′UTRSLI were significantly higher than those of rMM 1775, and the peak titer gap reached 15-fold at 60 dpi (Fig. 6E). The above data showed that the SLI element of the CQW1 3′UTR substitution significantly improved the growth ability of rMM 1775 in DEF cells, which suggested that the SLI structure of the duck TMUV 3′UTR played an important role in duck host-cell adaptation.

### Substitution of the 3′UTR SLI structure of MM 1775 and CQW1 attenuated the pathogenicity in mice.

**FIG 7:**
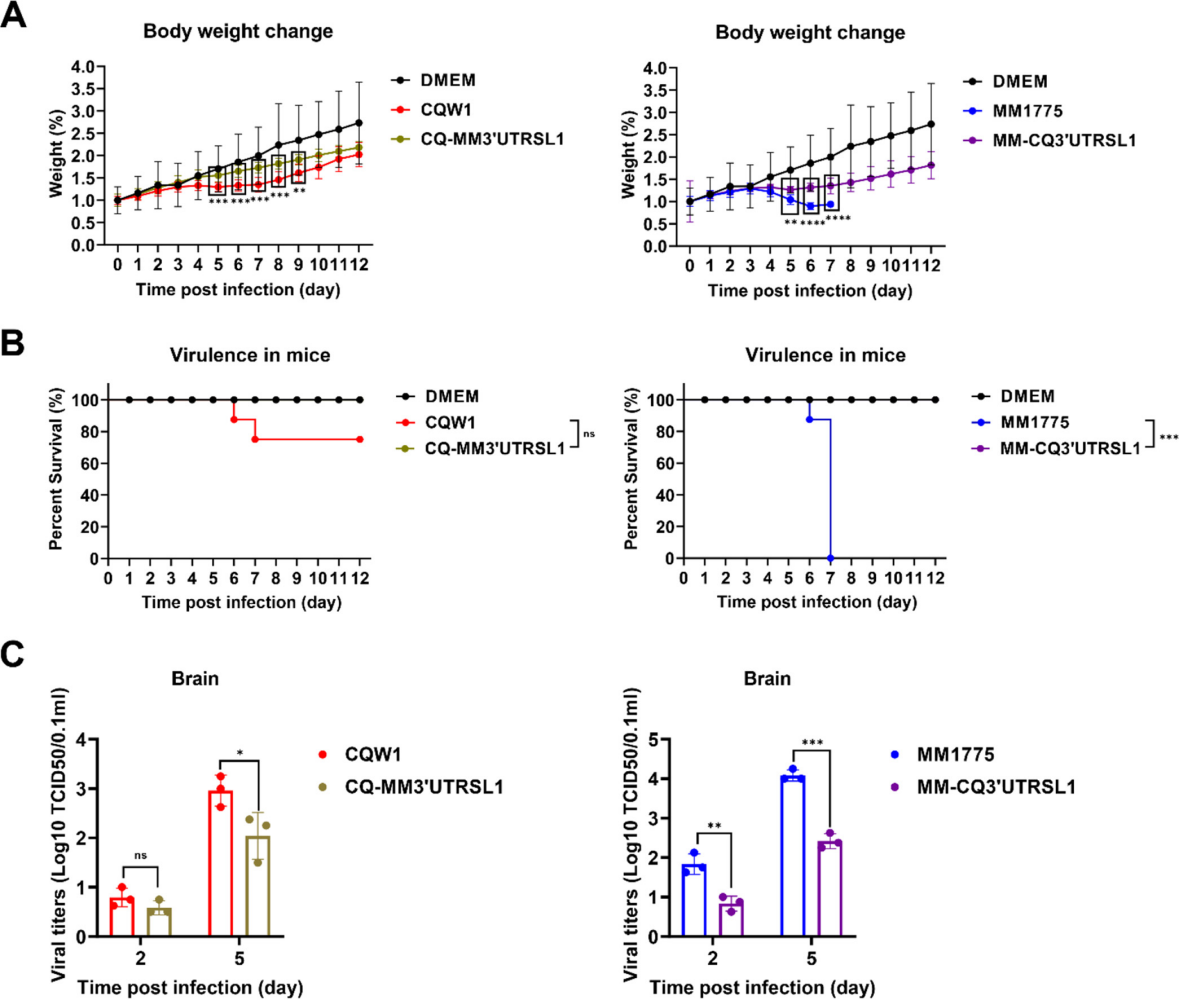
Pathogenicity of SLI chimeric viruses in mice. Fourteen-day-old Kunming mice were intracranially injected with parent or SLI chimeric viruses at 10^4.2^ TCID_50_/50 μL, respectively. (A) Weight change postinfection (days 0 to 12). Statistically significant differences in weight change at each time point were analyzed using multiple *t* tests with the Holm-Sidak method. (B) Survival curves of mice. Statistically significant differences in survival were analyzed using the log-rank (Mantel-Cox) test. (C) Viral titers in mouse brains at 2 and 5 dpi. The data represent the means and SDs of three independent individuals and were tested for statistical significance using Student’s *t* test. Statistical significance: *, *P* < 0.05; **, *P* < 0.01; ***, *P* < 0.001; ****, *P* < 0.0001; ns, no significance.

Finally, we further dissected the effect of the SLI element on viral pathogenicity in mice, and 14-day-old Kunming mice were infected with parent viruses or SLI chimeric viruses (rCQ-MM3′UTRSLI and rMM-CQ3′UTRSLI) at 10^4.2^ TCID_50_/50 μL by intracranial inoculation. To our surprise, both chimeric viruses exhibited attenuated virulence in mice. The weight of mice infected with rCQ-MM3′UTRSLI continuously increased, exhibiting a significant difference compared with CQW1-infected mice (Fig. 7A). Relevantly, mortality examination showed that mice in the rCQW1-infected group had 25% mortality, but no mice died in the rCQ-MM3′UTRSLI-infected group (Fig. 7B). In addition, viral titer measurements showed that at 5 dpi, CQW1-infected mice had an 8-fold higher viral titer in the brain than rCQ-MM3UTR’SLI-infected mice (Fig. 7C). While mice infected with rMM 1775 showed weight loss from 3 dpi and never recovered, with all mice dying within 7 days, mice in the rMM-CQ3′UTRSLI-infected group began to show a decline in weight from 5 dpi and recovery beginning at 8 dpi (Fig. 7A), and all mice survived for 12 days (Fig. 7B). Moreover, mice infected with rMM 1775 exhibited 10- or 46-fold higher viral titers at 2 and 5 dpi than mice infected with rMM-CQ3′UTRSLI (Fig. 7C).

In conclusion, the SLI element within the 3′UTR was an important factor in regulating viral virulence in mice for both rMM 1775 and rCQW1. After substitution, the virulence of both strains was notably attenuated.

### Partial deletion of the CQW1 SLI structure attenuated viral pathogenicity in mice with no effect on viral proliferation *in vitro*.

**FIG 8:**
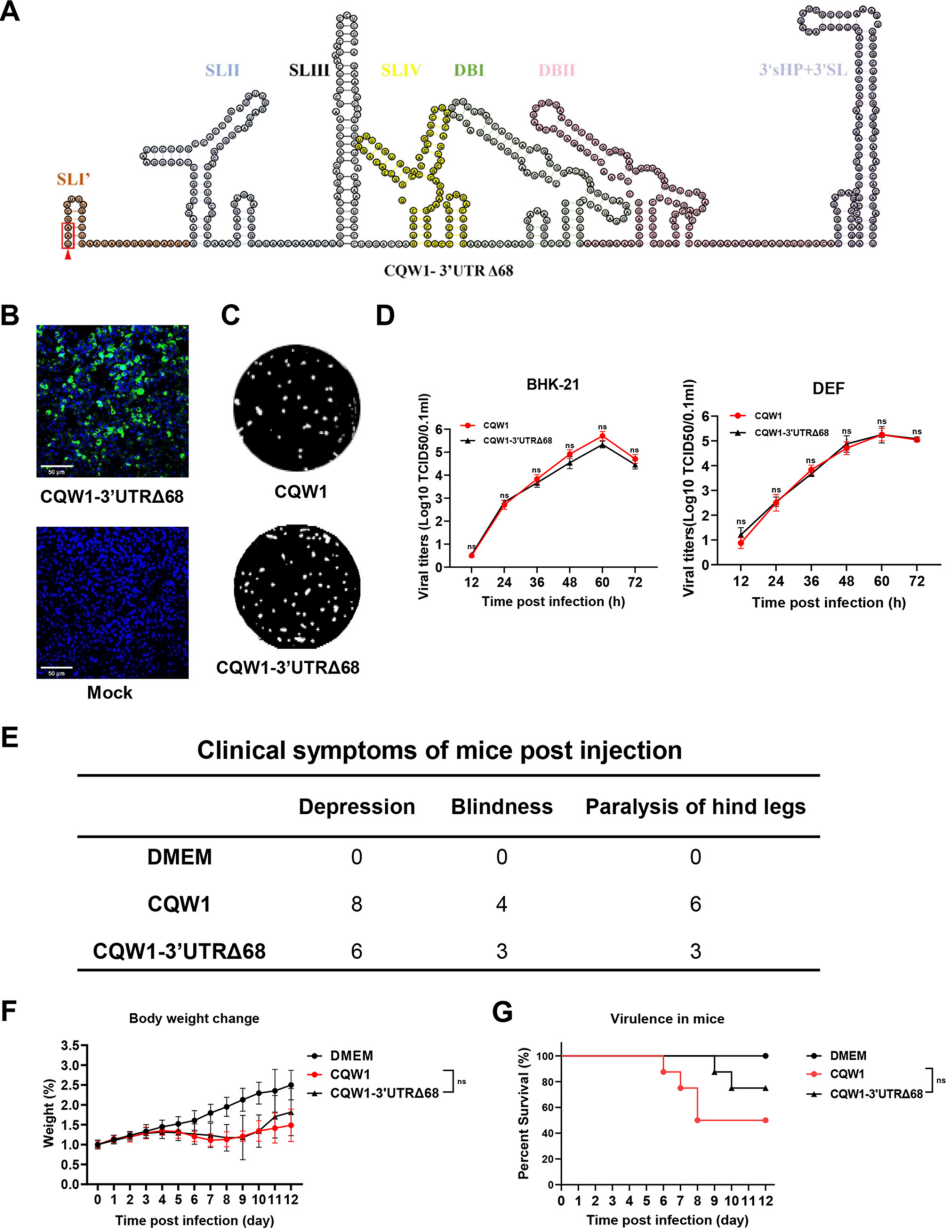
*In vitro* characteristics and pathogenicity of the CQW1 SLI mutant virus in mice. (A) RNA secondary structure prediction of SLI mutant virus (rCQW1-3′UTRΔ68). The stop codon UAA is highlighted in a red frame. (B) Recovery of rCQW1-3′UTRΔ68 confirmed by IFA in BHK-21 cells. (C) Plaque phenotypes of SLI mutant virus and CQW1. (D) Comparison of the growth kinetics of SLI mutant virus and rCQW1 in BHK21 cells (left) and DEF cells (right). All data represent the means and SDs of three independent cell cultures and were tested for statistical significance using Student’s *t* test. (E to G) Fourteen-day-old Kunming mice were intracranially infected with 10^4.375^/50 μL SLI mutant virus or parent virus, and clinical signs (E), weight changes (F), and mortality (G) were monitored daily. Statistical significance: ns, no significance.

As reported previously, the TMUV 3′UTR SLI spontaneously deleted 68 nucleotides (nt) after a series of passages in chicken embryos or chicken-origin DF-1 cells (26, 27). Hence, we further constructed and rescued rCQW1-3′UTRΔ68 (deletion the first 68 nt of the 3′UTR) to better understand its *in vitro* characteristics and virulence in mice (Fig. 8A and B). The rCQW1 and rCQW1-3′UTR SLIΔ68 showed similar plaque sizes in BHK-21 cells (Fig. 8C) and comparable growth kinetics in both BHK-21 cells and DEF cells (Fig. 8D). These data suggested that the first 68 nt of rCQW1 SLI might be redundant for viral *in vitro* proliferation in BHK-21 and DEF cells. Next, 14-day-old Kunming mice were intracranially infected with 10^4.375^/50 μL TCID_50_ rCQW1-3′UTR SLIΔ68 to evaluate its pathogenicity in mice. In contrast with the mice in the rCQW1-3′UTRΔ68-infected group, mice inoculated with rCQW1 exhibited more serious neurological symptoms (Fig. 8E) and similar weight changes (Fig. 8F), with mortality reaching 50% (4 of 8), while the mortality of mice in the former was only 25% (2 of 8) (Fig. 8G). This result indicated that the deletion of the first 68 nt in the SLI element attenuated the pathogenicity of rCQW1 in mice. All of the above data indicated that the partial deletion (first 68 nt) within CQW1 SLI conferred no effect on viral proliferation in BHK-21 and DEF cells but attenuated pathogenicity in 14-day-old Kunming mice.

## DISCUSSION

Outbreaks of flaviviruses cause widespread concern worldwide (28–31). As the reservoir host, birds are regarded as one of the major contributors to these outbreaks. In 1999, West Nile virus (WNV) reached North America and broke out, and huge numbers of deaths in crows, jays, and hawks were recorded during the epidemic. Later on, because of the increased range of the virus, the number of human cases also continued to rise (28, 32). In 2001, Usutu virus (USUV), a mosquito-borne flavivirus, was first reported to infect birds in Europe (33); in 2015 to 2016, widespread USUV activity was reported in Germany, France, Austria, Belgium, and The Netherlands, with mortality observed in blackbirds and gray owls (34). Meanwhile, many reports have indicated that humans can be infected with USUV as well, and the symptoms vary from moderate (e.g., rash, fever, and headache) to severe signs of infection (e.g., neurological disorders) (35–37). In April 2010, duck TMUV was first identified as the causative agent of duck egg-drop disease in China, and the infection and morbidity rates are typically high (up to 90%) (9, 13, 38). TMUV exhibits a wide range of bird host species, including chickens, ducks, geese, pigeons, and sparrows (39, 40), and most of them are important for the avian industry. To date, many studies have focused on molecular determinants, which are responsible for the broad spread of flaviviruses in birds, and several viral proteins have been demonstrated to play a key role in this aspect. While the T249P mutation in the WNV NS3 protein confers increased viral virulence in American crows due to enhanced replication, the NS1-2B protein modulates viral temperature sensitivity in avian leukocytes (41, 42). Additionally, WNV with E protein *N*-glycosylation shows higher pathogenicity in chickens and enhanced growth kinetics in avian cells but has no effect in mosquito cells, indicating that E-protein glycosylation may be a requirement for efficient transmission of WNV from avian hosts to mosquito vectors (43, 44). With its emergence in southeastern China, TMUV has caused huge economic losses in the duck industry. Some efforts were made to understand the outbreak of TMUV in ducks. Yan et al. found that the 156S in the E protein is critical for TMUV tissue tropism and transmissibility in ducks, while there is a P amino acid in MM 1775 at the same position to limit its horizontal transmission in ducks (45). However, few studies have investigated the host-specific adaptation function of the TMUV 3′UTR.

Host-specific adaptation is one of the necessary steps for viral host switching. In the present study, we first verified that the duck-origin strain rCQW1 exhibited higher cellular fitness in DEF cells and BHK-21 cells than the mosquito-origin strain rMM 1775, and the results were the same as those previously reported (24). Then, we further found that duck-derived CQW1 3'UTR could improve the proliferation ability of mosquito-derived rMM 1775 in BHK-21 and DEF cells. Therefore, the TMUV 3′UTR was relevant to viral host-specific adaptation. To date, many studies have indicated that the flavivirus 3′UTR plays a crucial role in viral host-specific adaptation. Using DENV as a model, after a series of passages restricted in mosquito cells, a large number of virus populations with various SLII structures were observed, and recombinant DENV carrying the mutated SLII structure showed higher viral fitness in mosquito cells than the parent DENV with a slight influence on viral replication in human cells (18). In addition, point mutations in DENV 3′sHP abrogated viral infection in mosquito cells without affecting replication in mammalian cells (46). Moreover, ZIKV and DENV evolved functionally equivalent SL structures in inverted locations of their 3′UTRs. In this regard, the lack of either SLI or SLII slightly reduced viral replication in human cells, and the deletion of SLI resulted in higher replication in mosquito cells (19). In JEV, partial deletions of SL or DB structures had no apparent effects on virus replication in either mammalian or mosquito cells (47). These results also suggest the presence of different evolutionary strategies for host-specific fitness among flaviviruses. Partial deletion mapped in the SLI structure of duck-derived TMUV is detected after a series of passages in DF-1 cells or chicken embryos, indicating that the structure may be related to host adaptation (26, 27). In fact, we observed that neither the 3′UTR nor the SLI structure had little influence on the efficient proliferation in mammalian cells and avian cells for duck-origin TMUV. Therefore, we assumed that other regions of the duck-derived TMUV genome may be important for viral cellular proliferation, such as the envelope protein. Sun et al. discovered that the position 367 played an important role for viral proliferation in DEF and BHK-21 cells (48). In addition, recombinant rMM 1775 carrying the SLI element from CQW1 exhibited higher proliferation ability in BHK-21 and DEF cells, suggesting that the 3'UTR was a key factor to viral host-specific adaptation for mosquito-borne TMUV. However, these results also supposed that the 3'UTR of TMUV might not be a determinant of viral cellular fitness in the evolution from mosquito to duck adaption. In fact, for many multihost viruses (e.g., influenza virus and coronavirus), the cross-species transmission is a complex process, and many factors may contribute to this process, instead of only a single factor that plays a predominant role (2, 49).

Here, we confirmed that TMUV was able to effectively infect Kunming mice via intracranial injection, and rMM 1775 exhibited stronger pathogenicity in mice than rCQW1 as reflected by the weight changes, mortalities, and viral loads in the brain. A DENV-3 vaccine candidate carrying DENV-4 3′UTR with a 30-nucleotide deletion exhibited highly attenuated virulence in mice (50). In TBEV, by substation mutations, researchers found that the variable region of the 3′ UTR was a critical virulence factor in the far-eastern subtype of tick-borne encephalitis virus in the C57BL6 mice (51). Both of these results indicated that the flavivirus 3′UTR might be a crucial factor in regulating the pathogenicity of viruses in mice. Here, we found that recombinant MM 1775 carrying the 3′UTR from CQW1 displayed attenuated viral pathogenicity in mice as well, but for CQW1, the entire substitution of the 3′UTR from MM 1775 showed a slight impact on viral pathogenicity in mice. Thus, it appears that 3'UTR from different TMUV strains maintains diverse functions in virulence in mice; while the 3′UTR is essential for effective infection of mosquito-derived TMUV, it plays a redundant function for duck-derived TMUV. We further discovered that whether it was the substitution or deletion mutation or substitution with duck-derived SLI or mosquito-derived SLI, the virulence of the recombinant virus was weakened. These results suggested that the SLI element was relevant to viral pathogenicity in mice and could be explained by the fact that the matching of SLI and the genome of the virus affected the characteristics of the TMUV, which has been found to be common in flaviviruses. For example, in ZIKV, the last 285 nucleotides of 3′UTR RNA are responsible for binding to NS2A protein to mediate virus assembly (52). In DENV, the sequences within DBI hybridized with a region present in the capsid coding sequence, and disassembling DBI structure promoted long-range interactions and genome cyclization (17). In JEV, some mutations located at the SLIV and DBI regions are related to viral proliferation and pathogenicity. All of the above-mentioned findings suggest that the flavivirus 3′UTR is an important factor that is relevant to viral pathogenicity in mice.

Taken together, by studying the biological characteristics of chimeric viruses, the relevance of the SLI structure within the TMUV 3′UTR in host-specific adaptation between mosquitos and ducks was discovered for the first time. Additionally, we also observed that the SLI structure is one of the factors regulating the neurovirulence of TMUV in mice. However, the molecular mechanisms of the key determinants of interspecies transmission of TMUV from mosquito to duck remain unclear. In the future, we will continue to concentrate on the role of the TMUV 3′UTR in cross-species transmission.

## MATERIALS AND METHODS

### Ethics statement.

All mouse experimental procedures were approved by the Institutional Animal Care and Use Committee of Sichuan Agriculture University in Sichuan, China, under protocol permit number SYXK(川)2019-187.

### Cells and viruses.

TMUV infections were performed using the following cell lines. C6/36 cells, an Aedes albopictus cell line gifted by Rui Luo, Huazhong Agricultural University, were cultured in RPMI medium 1640 basic (Gibco, Shanghai, China) supplemented with 10% fetal bovine serum (FBS) (Gibco, New York, USA) and 1% ciprofloxacin and incubated at 28°C with no additional CO_2_. BHK-21 cells were cultured in Dulbecco’s modified Eagle’s medium (DMEM) (Gibco, Shanghai, China) supplemented with 10% FBS and 1% ciprofloxacin and incubated at 37°C with 5% CO_2_. DEF cells were isolated from 9-day-old duck embryos, cultured in DMEM supplemented with 10% newborn calf serum (NBCS) (Gibco Shanghai China) and 1% ciprofloxacin, and incubated at 37°C with 5% CO_2_.

The duck-derived TMUV strain CQW1 (GenBank ID KM233707.1) was rescued from a DNA-based full-length infectious clone reported previously (53). The mosquito-derived TMUV strain MM 1775 (GenBank ID JX477685.2) was collected as mentioned above (24). All virus stocks were amplified in BHK-21 cells.

### Viral titration and Western blotting.

The viral titer was determined according to the median tissue culture infectious dose (TCID_50_) method and calculated by the Karber method as reported previously (23). BHK-21 or DEF cells were infected with rCQW1 or rMM 1775 at 300 TCID_50_. After 24 or 48 h, the supernatant was removed, and the cells were harvested and analyzed by Western blotting. A mouse anti-DTMUV-NS3 polyclonal antibody (self-prepared) and a mouse anti-β-actin antibody (Ruiying Biological, China) were used as primary antibodies. Horseradish peroxidase (HRP)-conjugated goat anti-mouse IgG (Abcam, Shanghai, China) was used as the secondary antibody. The proteins were visualized using Clarity Western ECL Substrate (Bio-Rad, USA).

### Sequence alignment and RNA secondary prediction.

The 3′UTR nucleotide pairwise alignment of CQW1 and MM 1775 was measured using Geneious software with ClustalW algorithms. Nucleotide similarity was analyzed using DNAMAN software. The RNA secondary structures of the CQW1 and MM 1775 3′UTRs were predicted using the RNAfold web server (http://rna.tbi.univie.ac.at/cgi-bin/RNAWebSuite/RNAfold.cgi), UNAFold web server (http://www.unafold.org/), and VARNAv software and modified artificially. Twenty available TMUV 3′UTR sequences were obtained from the NCBI database to perform phylogenetic analysis using MEGA7 software with the neighbor-joining (NJ) method.

### Plasmid construction and recombinant virus rescue.

Two stable DNA-based full-length infectious clones, pACNR-MM 1775-Intron and pACNR-CQW1-Intron (24, 53), were used to construct all five recombinant plasmids. Briefly, to construct pACNR-MM-CQ3′UTR and pACNR-MM-CQ3′UTRSLI, corresponding substituted mutational fragments were amplified by a series of overlapping PCRs and assembled in pACNR-MM 1775 Intron by exploiting the restriction endonucleases EcoRI and NruI (NEB, Beijing, China). At the same time, to construct pACNR-CQ-MM3′UTR, pACNR-CQ-MM3′UTRSLI, and pACNR-CQW1-3′UTRΔ68, corresponding substituted or SLI-deleted mutational fragments were amplified by a series of overlapping PCRs and assembled into pACNR-CQW1 Intron by using the restriction endonucleases EcoRI and NruI (NEB, Beijing, China). The primers used in this study are shown in Table 1.

**TABLE 1:** Primers used in this study for overlapping PCR

Name	Sequence (5′ to 3′)
CQW1-SbfI-F	CCCAGAACCACCTGCAGG
CQW1-NS5-R	TTACAAAACACCTTCACTCCAGCT
CQ-MM3'UTR-F	AGTGAAGGTGTTTTGTAAATATATGAAATAGGAGTAGAATGTAAATAAAGTAG
TMUV-NruI-R	TCAACGGGAAACGTCTTGTCGCGATAAGATACATTGATGAGTT
MM1775-EcoRI-F	AGCAGTTGAAGATCCAGAATTC
MM1775-NS5-R	TTACAAGACGCCCTCACTCCA
MM-CQ3'UTR-F	GAGGGCGTCTTGTAAATATATGAGGTAGGTGTAAAAATGTATGTAAAG
TMUV-SLIsub-F	GGAAGTCAGGCCAGGGAATC
TMUV-SLIsub-R	GATTCCCTGGCCTGACTTCC
CQW1-3′UTRΔ68-F	AGTGAAGGTGTTTTGTAAGCATTTGTTTGAATAGATAGGAAGAG

To rescue recombinant viruses, BHK-21 cells were seeded in 12-well plates, and when the cells were 70 to 90% confluent, they were transfected with 1.6 μg of recombinant plasmids using TransIntro EL transfection reagent (TransGen Biotech, Beijing, China) according to the manufacturer’s instructions. After transfection for 72 h, the supernatant (F0 virus) was harvested and used to infect fresh BHK-21 cells again until a clear cytopathic effect (CPE) appeared. Then, the supernatant (F1 virus) was collected, aliquoted, and stored at −80°C.

### Indirect immunofluorescence assay (IFA).

BHK-21 cells were seeded in 12-well plates and infected with recombinant viruses at 400 TCID_50_. At approximately 60 h postinfection, the supernatant was removed, and the cells were washed with phosphate-buffered saline (PBS), fixed with 4% paraformaldehyde for 1 h, permeabilized for 40 min at 4°C with 0.3% Triton in PBS, and blocked with 5% bovine serum albumin (BSA) in PBS for 1 h at 37°C. The cells were washed three times with 0.1% PBS/Tween 20 (PBST) after each step. Thereafter, the cells were treated with mouse anti-TMUV polyclonal antibody (self-prepared) as the primary antibody at 4°C overnight. Then, the cells were washed with PBST and incubated with fluorescein isothiocyanate (FITC)-labeled goat anti-mouse IgG (Beyotime Biotech, Beijing, China) as the secondary antibody for 1 h at 37°C, after which the nucleotides were counterstained with 4’,6-diamidino-2-phenylindole (DAPI; Solarbio, China) for 20 min at room temperature. Finally, the cells were imaged using a fluorescence microscope (Nikon, Japan).

### Growth curve.

To test the viral proliferation properties *in vitro*, C6/36, DEF, and BHK-21 cells were seeded in 24-well plates. When the cells reached 70 to 90% confluence, the supernatant was removed, and the cells were washed twice with PBS and infected with recombinant viruses at 250 TCID_50_ (23, 24). After 1.5 h of attachment at 28 or 37°C, the inocula were removed. Next, the cells were washed twice with PBS and supplemented with RPMI 1640 basic medium or DMEM containing 2% FBS and 1% ciprofloxacin. The supernatant was harvested every 12 h and subjected to viral titration.

### Plaque assay.

Plaque assays were performed as described previously (23). Recombinant or parent viruses were serially diluted 10-fold in DMEM; 400 μL of viruses of each dilution was added to a 12-well plate seeded with BHK-21 cells at approximately 95% confluence. After 1.5 h of attachment at 37°C, 1 mL of 1% methyl cellulose overlay containing 2% FBS and 1% ciprofloxacin was added to each well, and the plate was incubated for 6 days. Subsequently, the overlay was removed, and the plate was washed twice with PBS, fixed with 4% formaldehyde at room temperature for 20 min, and then stained with 1% crystal violet for 1 min. Finally, the cells were washed carefully, and visible plaques were observed.

### Duck embryo virulence.

The duck embryos were purchased from the Waterfowl Breeding Center of Sichuan Agriculture University. Nine-day-old duck embryos were divided into four experimental groups (*n* = 10/group) and a mock group. Embryos in the experimental groups were inoculated with 1,000 TCID_50_ of parent or recombinant viruses, respectively, in a volume of 100 μL via the allantoic cavity route (24, 54). Additionally, the mock group was injected with DMEM using the same route and volume. All embryos were from Peking ducks and hatched in the avian incubator-HX-176 (Beili Chengdu) at 37°C for 7 days during this study. The mortality of the embryos was monitored and recorded daily.

### Animal experiments.

To determine viral pathogenicity in mice, 14-day-old Kunming mice were randomly divided into two parts. In the first part, the mice were divided into experimental and mock groups (*n* = 8/group). The mice in the experimental groups were injected with 50 μL parent or recombinant virus by intracranial inoculation at 10^4.375^ TCID_50_/50 μL or 10^4.2^ TCID_50_/50 μL (55). The mice in the mock group were injected with DMEM using the same route and volume. Mouse weight changes, clinical signs, and mortality were monitored and recorded for 12 days or until the number of mice was less than three. In the other part (*n* = 6/group), the mice were treated in the same way as described above. Three mice in each group were euthanized and necropsied at 2 and 5 dpi. The brains were collected to determine the viral titers by TCID_50_ in BHK-21 cells.

### Statistical analysis.

All data were analyzed using GraphPad Prism 8.0 (La Jolla, CA, USA). The data for the viral titers are presented as the means ± standard errors (SEM). Student’s *t* test was used to assess statistical significance. The statistical significance of duck embryo survival was analyzed using a survival curve and the log-rank (Mantel-Cox) test. The data on mouse weight changes were analyzed based on multiple *t* tests with the Holm-Sidak method, and all of the above statistical significance levels were defined by a *P* value of <0.05 (*).

### Data availability.

The raw data supporting the conclusions of this article will be made available by the authors, without undue reservation.
